# miR-4516-Loaded Engineered Milk Extracellular Vesicles Attenuate Indoxyl Sulfate-Induced Mitochondrial Dysfunction and Improve Renal Function in a CKD Mouse Model

**DOI:** 10.3390/ijms27072997

**Published:** 2026-03-25

**Authors:** Jeongkun Lee, Jun Young Yoon, Jae Young Lee, Sang Hun Lee

**Affiliations:** Department of Biomedical Sciences, College of Medicine, Program in Biomedical Science & Engineering, Inha University, 3-ga, Sinheung-dong, Jung gu, Incheon 22332, Republic of Korea; jeongkun@inha.ac.kr (J.L.); yoon981008@inha.edu (J.Y.Y.); brookin@inha.edu (J.Y.L.)

**Keywords:** chronic kidney disease, milk-derived extracellular vesicles, miR-4516, indoxyl sulfate, mitochondrial dysfunction

## Abstract

Chronic kidney disease (CKD) involves uremic toxin-driven tubular injury and systemic vascular dysfunction, in which mitochondrial impairment and apoptotic cell loss contribute to progressive tissue deterioration. Accordingly, a targeted EV platform is required to enable efficient miRNA delivery to the toxin-stressed tubular–endothelial compartment. Based on our previous study showing that melatonin restores miR-4516 levels under CKD-related stress, we directly loaded miR-4516 into engineered extracellular vesicles (EVs) to evaluate its effects on mitochondrial function and cell survival. Here, we engineered EVs with a G3-C12/RGD surface modification and established a miR-4516 loading strategy to enhance delivery to kidney proximal tubule cells and vascular endothelial cells. miR-4516 loading increased EV-associated miR-4516 levels without major changes in particle size distribution, and EV identity was supported by CD9 and CD81 expression. Confocal microscopy and flow cytometry demonstrated increased cellular uptake of miR-4516-loaded G3-C12/RGD-EVs compared with control EVs in TH1 proximal tubule cells and HUVECs. Under indoxyl sulfate stress, engineered EV treatment restored intracellular miR-4516 and improved mitochondrial function, as indicated by recovery of respiratory Complex I and Complex IV activities and improved Seahorse bioenergetic parameters (OCR/ECAR, basal and maximal respiration, ATP-linked respiration, and spare respiratory capacity). Annexin V staining further indicated reduced toxin-induced apoptosis. In an adenine diet-induced CKD mouse model, intravenous administration of miR-4516-loaded G3-C12/RGD-EVs improved urinary albumin-to-creatinine ratio (UACR), blood urea nitrogen (BUN), and serum creatinine. These findings indicate that miR-4516-loaded, targeting-engineered EVs may mitigate uremic toxin-associated mitochondrial dysfunction and renal impairment in CKD.

## 1. Introduction

Chronic kidney disease (CKD) is defined as persistent abnormalities of kidney structure or function for at least 3 months and is a major contributor to global morbidity and mortality [1]. Global burden studies report a sustained increase in CKD-related deaths over recent decades, indicating a continued need for interventions that slow progression and address pathogenic processes leading to loss of renal function [2]. Tubulointerstitial fibrosis is a key determinant of irreversible decline, characterized by excessive extracellular matrix deposition and architectural distortion that compromise nephron structure and function [3]. Importantly, cellular stress responses that preceded overt fibrosis—including mitochondrial impairment and injury-associated cell loss in tubular epithelium and the vasculature—can promote maladaptive remodeling and functional deterioration [3,4].

At the cellular level, fibrotic progression is closely linked to maladaptive stress responses in renal tubular epithelial cells and their interactions with the surrounding microenvironment [3,4]. Uremic retention solutes contribute to this injury landscape and extend pathological effects to the vasculature, reinforcing endothelial dysfunction and adverse cardiorenal coupling in CKD [5,6]. Indoxyl sulfate, a protein-bound uremic toxin, promotes pro-inflammatory and profibrotic programs in proximal tubular cells, including activation of fibrotic signaling and extracellular matrix-related pathways [7,8]. Uremic toxin exposure is also associated with mitochondrial dysfunction and impaired bioenergetics, which can amplify tubular injury and favor fibrogenic remodeling [3]. These intracellular disturbances influence renal tubule–endothelium crosstalk through altered paracrine signaling, endothelial impairment, and microvascular rarefaction, forming a self-reinforcing cycle that sustains hypoxia and fibrosis [4,6,9].

MicroRNAs (miRNAs) are post-transcriptional regulators that coordinate stress responses relevant to cytoskeletal extracellular remodeling, metabolic dysfunction, and fibrosis-associated signaling [3]. miRNAs that modulate both cytoskeletal organization and mitochondrial integrity are of interest because they may influence multiple fibrosis-linked axes. In our previous study, miR-4516 was reduced under CKD/uremic toxin-related stress, and melatonin treatment restored miR-4516 levels, accompanied by improved mitochondrial homeostasis and attenuation of renal fibrotic remodeling [10]. These findings provide a rationale to evaluate miR-4516 supplementation via direct delivery in CKD. However, therapeutic implementation of miRNA modulation remains constrained by delivery barriers, off-target distribution, and limited accumulation within disease-relevant renal and vascular compartments [11,12,13,14]. Moreover, delivery platforms that can efficiently engage both tubular and endothelial compartments and provide functional rescue under uremic toxin stress remain limited.

Extracellular vehicles (EVs) have been investigated as carriers for intercellular communication and as delivery vehicles for nucleic acids, given their membrane encapsulation and biocompatibility [12,13]. Nevertheless, achieving targeted delivery and consistent cellular uptake in defined cell populations within complex tissues remains challenging, and harmonized reporting and characterization have been emphasized to support reproducibility and translational development [11,13]. Engineering strategies such as surface modification and ligand display have been studied to enhance tissue or cell-type tropism and improve delivery efficiency [11,13]. Among ligand-based approaches, the Arg–Gly–Asp (RGD) motif is a recognition sequence for several RGD-binding integrins and has been widely used to promote binding to integrin-enriched endothelium under pathological conditions [14,15].

In the present study, we addressed renal injury under uremic toxin conditions by focusing on the renal tubule–endothelium axis, given that tubular epithelial dysfunction and endothelial impairment can reinforce each other and accelerate CKD progression. We engineered extracellular vesicles (EVs)via G3-C12/RGD surface modification to enhance cellular association/uptake in both renal proximal tubule cells (TH1) and vascular endothelium (HUVECs) and generated a miR-4516-loaded formulation to evaluate a mitochondria-relevant miRNA cargo in this setting. We confirmed engineered EV properties and miR-4516 loading, and quantified cellular association/uptake using confocal microscopy and flow cytometry. Under indoxyl sulfate stress, we examined whether miR-4516 delivery is associated with partial restoration of mitochondrial bioenergetics and attenuation of cellular injury by integrating respiratory complex activity measurements, Seahorse-derived OCR/ECAR indices, and apoptosis readouts. Finally, we assessed whether intravenous administration of engineered EVs improves renal functional indices (UACR, BUN, and serum creatinine) in CKD mice.

## 2. Results

### 2.1. Characterization of Engineered Milk-Derived Extracellular Vesicles Loaded with miR-4516

**Figure 1:**
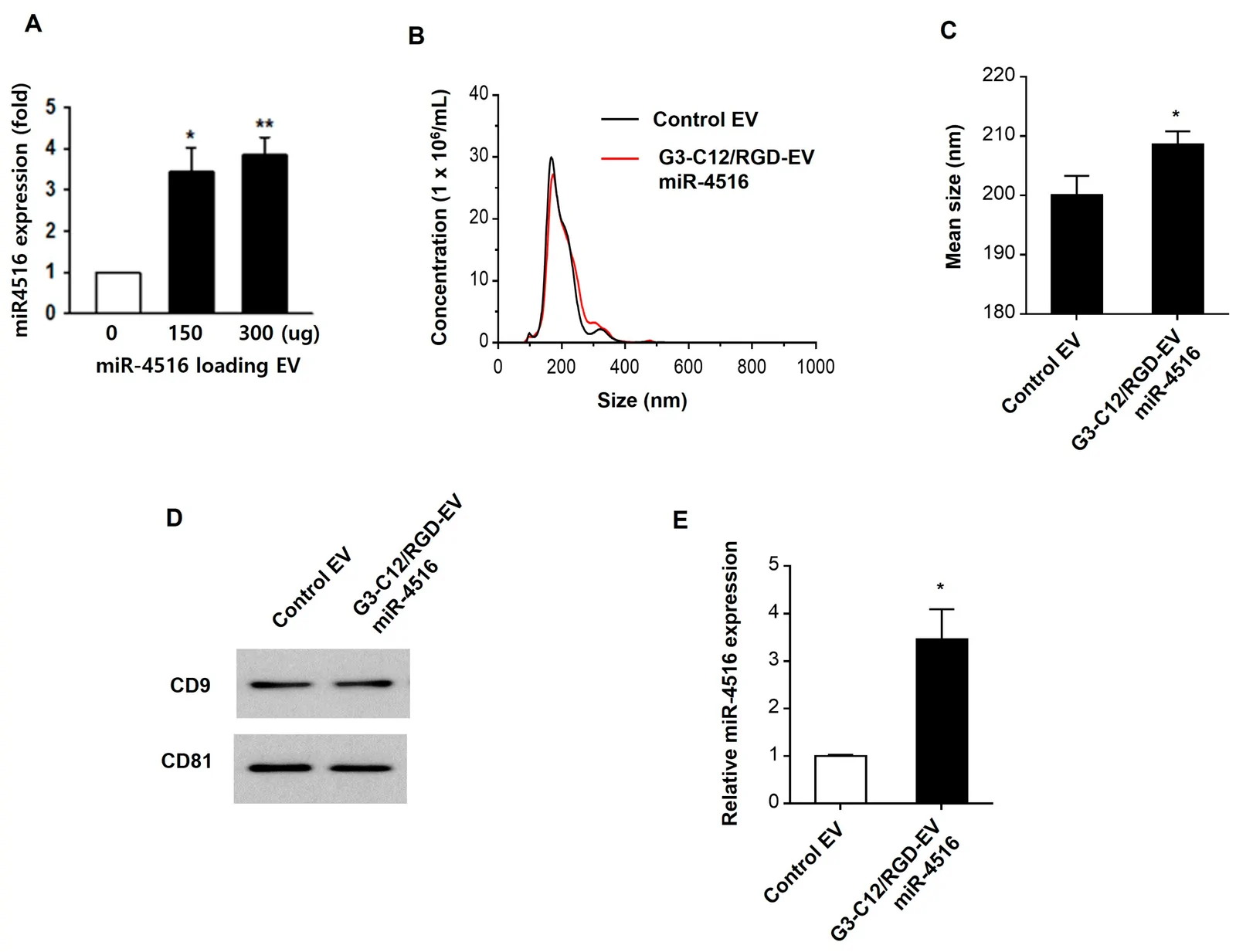
Characterization of miR-4516-loaded G3-C12/RGD-engineered EVs. (**A**) RT–qPCR quantification of miR-4516 in EVs loaded with 0, 150, or 300 µg miR-4516. (**B**) NTA size distribution of control EV and miR-4516-loaded G3-C12/RGD-EVs. (**C**) Mean particle size from NTA. (**D**) Immunoblots for CD9 and CD81. (**E**) Relative miR-4516 expression in miR-4516-loaded G3-C12/RGD-EVs versus control EVs by RT–qPCR. Data are presented as mean ± SEM (*n* = 3 independent EV preparations). Statistics: one-way ANOVA with Dunnett’s post hoc test vs. 0 µg (**A**); two-tailed Student’s *t*-test vs. control EV (**C**,**E**). In (**A**), * *p* < 0.05 and ** *p* < 0.01 vs. 0 µg. In (**C**,**E**), * *p* < 0.05 vs. control EV.

To generate targeting-engineered EVs, milk-derived EVs were surface-modified with the G3-C12/RGD moiety (G3-C12/RGD-EVs; described in Methods) and subsequently loaded with miR-4516 (Figure 1). miR-4516 levels in EVs increased in a dose-dependent manner, with significantly higher expression observed in the 150 µg and 300 µg loading conditions compared with 0 µg (Figure 1A). Nanoparticle tracking analysis (NTA) indicated that the particle size distribution of miR-4516-loaded G3-C12/RGD-EVs remained broadly comparable to that of control EVs, showing a dominant population in the ~150–200 nm range (Figure 1B). The mean particle size was modest but significantly increased in miR-4516-loaded G3-C12/RGD-EVs relative to control EVs (Figure 1C). Immunoblotting confirmed the presence of EV markers CD9 and CD81 in both control EV and miR-4516-loaded G3-C12/RGD-EV preparations (Figure 1D). Consistently, quantitative analysis demonstrated a significant enrichment of miR-4516 in miR-4516-loaded G3-C12/RGD-EVs compared with control EVs (Figure 1E). Collectively, these data support the successful generation of targeting-engineered, miRNA-loaded EVs while maintaining EV marker expression and an overall similar size distribution profile.

### 2.2. RGD-Functionalized, miR-4516-Loaded Engineered EVs Show Increased Cellular Association/Uptake in TH1 Cells and HUVECs

**Figure 2:**
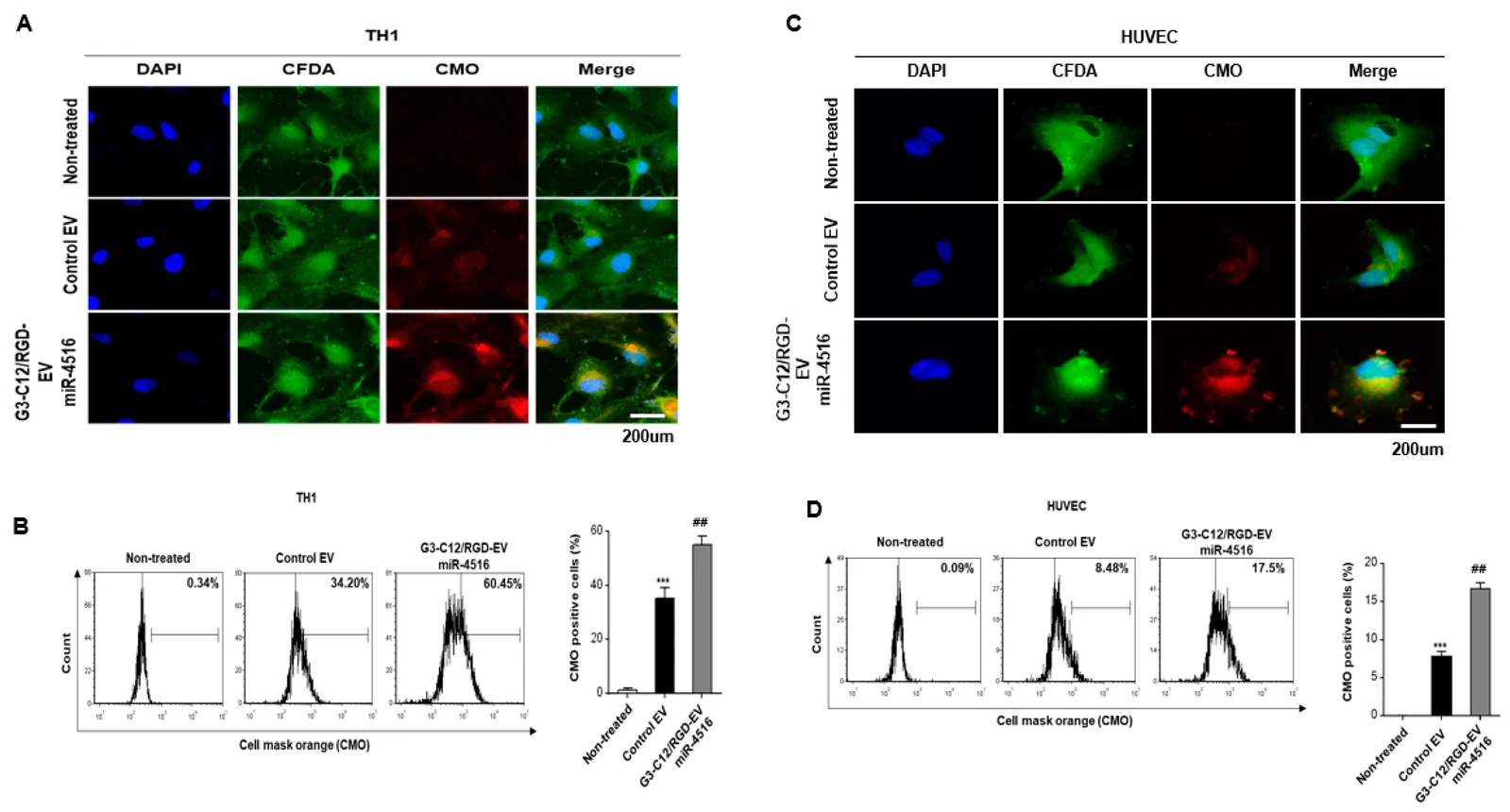
RGD-functionalized, miR-4516-loaded engineered EVs show increased cellular association/uptake in TH1 cells and HUVECs. (**A**) Representative fluorescence images of TH1 cells treated as indicated (Non-treated, Control EV, and G3-C12/RGD-EV miR-4516). Nuclei were stained with DAPI (blue), cells were labeled with CFDA (green), and EV membranes were labeled with CellMask Orange (CMO; red). Merged images are shown in the rightmost column. Scale bar is shown. (**B**) Flow cytometry analysis of CMO fluorescence in TH1 cells. Representative histograms (CMO-positive gate) and quantification of the percentage of CMO-positive cells are shown (non-treated, 0.34%; Control EV, 34.20%; G3-C12/RGD-EV miR-4516, 60.45%). *** *p* < 0.01 for Control EV vs. Non-treated; ## *p* < 0.01 for G3-C12/RGD-EV miR-4516 vs. Control EV. (**C**) Representative fluorescence images of HUVECs under the same conditions as in (**A**) with DAPI (blue), CFDA (green), and CMO (red). Merged images are shown. Scale bar is shown. (**D**) Flow cytometry analysis of CMO fluorescence in HUVECs with representative histograms and quantification of CMO-positive cells (non-treated, 0.09%; Control EV, 8.48%; G3-C12/RGD-EV miR-4516, 17.5%). *** *p* < 0.01 for Control EV vs. Non-treated; ## *p* < 0.01 for G3-C12/RGD-EV miR-4516 vs. Control EV.

EV membranes were labeled with CellMask Orange (CMO) to track EV cellular association/uptake. In TH1 cells, confocal microscopy showed minimal CMO signal in non-treated cells, whereas EV exposure produced clear CMO-associated fluorescence, which was more prominent with miR-4516-loaded G3-C12/RGD-engineered EVs than with control EVs (Figure 2A). These observations were supported by flow cytometry, where the fraction of CMO-positive TH1 cells increased from 0.34% (non-treated) to 34.20% (Control EV) and 60.45% (G3-C12/RGD-EV miR-4516) (Figure 2B). In HUVECs, confocal imaging similarly demonstrated low background CMO signals in the non-treated group and increased CMO-associated fluorescence after EV treatment, with a stronger signal observed in the G3-C12/RGD-EV miR-4516 condition compared with Control EV (Figure 2C). Consistently, flow cytometry quantified an increase in CMO-positive HUVECs from 0.09% (non-treated) to 8.48% (Control EV) and 17.5% (G3-C12/RGD-EV miR-4516) (Figure 2D), indicating enhanced EV cellular association/uptake in both proximal tubular cells and endothelial cells.

### 2.3. Engineered EV-Mediated miR-4516 Delivery Restores the miR-4516/SIAH3/PINK1 Axis and Improves Mitochondrial Respiratory Complex Activities in Indoxyl Sulfate-Treated TH1 Proximal Tubule Cells

**Figure 3:**
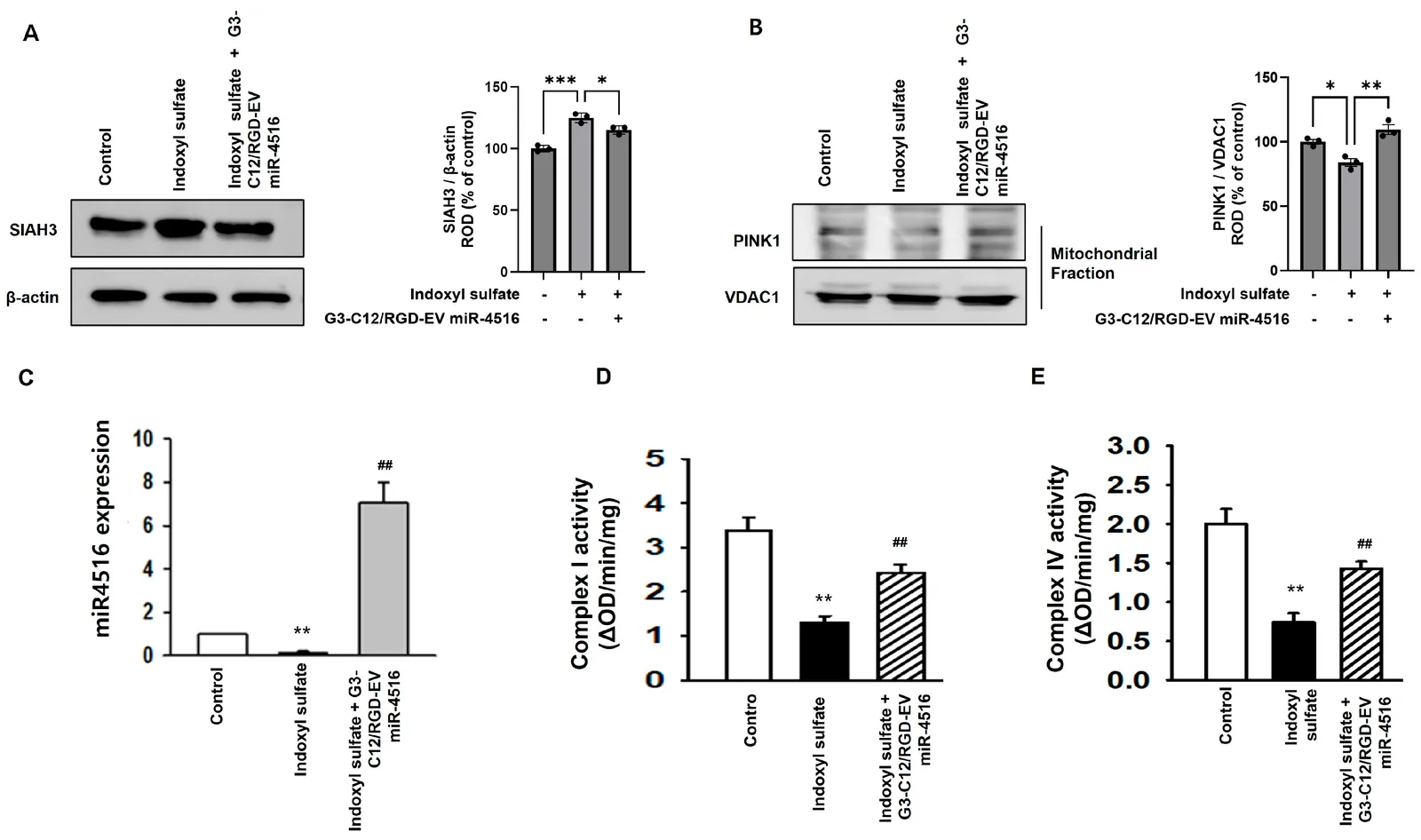
miR-4516 delivery by G3-C12/RGD-engineered EVs modulates the SIAH3/PINK1 axis and improves mitochondrial respiratory complex activities in indoxyl sulfate-treated TH1 proximal tubule cells. TH1 proximal tubule cells were exposed to indoxyl sulfate (IS) and then treated with miR-4516-loaded G3-C12/RGD-engineered EVs (G3-C12/RGD-EV miR-4516). (**A**) Representative Western blot images of SIAH3 and β-actin (**left**) and corresponding quantification of SIAH3/β-actin (**right**). (**B**) Representative Western blot images of mitochondrial PINK1 and VDAC1 (**left**) and corresponding quantification of PINK1/VDAC1 (**right**). (**C**) Intracellular miR-4516 expression under the indicated conditions. (**D**) Mitochondrial Complex I activity (ΔOD/min/mg). (**E**) Mitochondrial Complex IV activity (ΔOD/min/mg). Data are presented as mean ± SEM (n = 3). Statistical analysis was performed by one-way ANOVA followed by Tukey’s post hoc test. In (**A**), *** *p* < 0.001 vs. Control and * *p* < 0.05 vs. IS. In (**B**), * *p* < 0.05 vs. Control and ** *p* < 0.01 vs. IS. In (**C**–**E**), ** *p* < 0.01 vs. Control and ## *p* < 0.01 vs. IS.

To determine whether engineered EV-mediated miR-4516 delivery modulates mitochondrial regulatory signaling in indoxyl sulfate (IS)-treated TH1 proximal tubule cells, we first examined the expression of SIAH3 and mitochondrial PINK1. Western blot analysis showed that IS significantly increased SIAH3 expression compared with the control group, whereas treatment with G3-C12/RGD-EV miR-4516 reduced SIAH3 levels relative to IS alone (Figure 3A). In parallel, mitochondrial PINK1 levels were decreased by IS treatment, while G3-C12/RGD-EV miR-4516 restored mitochondrial PINK1 expression toward control levels (Figure 3B).

Consistent with these findings, IS markedly reduced intracellular miR-4516 expression, whereas treatment with G3-C12/RGD-EV miR-4516 significantly increased miR-4516 levels compared with IS alone (Figure 3C). We next assessed whether these molecular changes were associated with mitochondrial functional recovery. IS significantly decreased the activities of mitochondrial respiratory chain Complex I and Complex IV, indicating impaired oxidative phosphorylation. Importantly, treatment with G3-C12/RGD-EV miR-4516 significantly increased both Complex I and Complex IV activities compared with IS alone, although not fully to control levels (Figure 3D,E). Collectively, these findings indicate that engineered EV-mediated miR-4516 delivery attenuates IS-induced mitochondrial dysfunction, at least in part, through modulation of the miR-4516/SIAH3/PINK1 axis.

### 2.4. Seahorse Extracellular Flux Analysis Shows That miR-4516-Loaded G3-C12/RGD-Engineered EVs Partially Restore Mitochondrial Respiration Impaired by Indoxyl Sulfate in TH1 Proximal Tubule Cells

**Figure 4:**
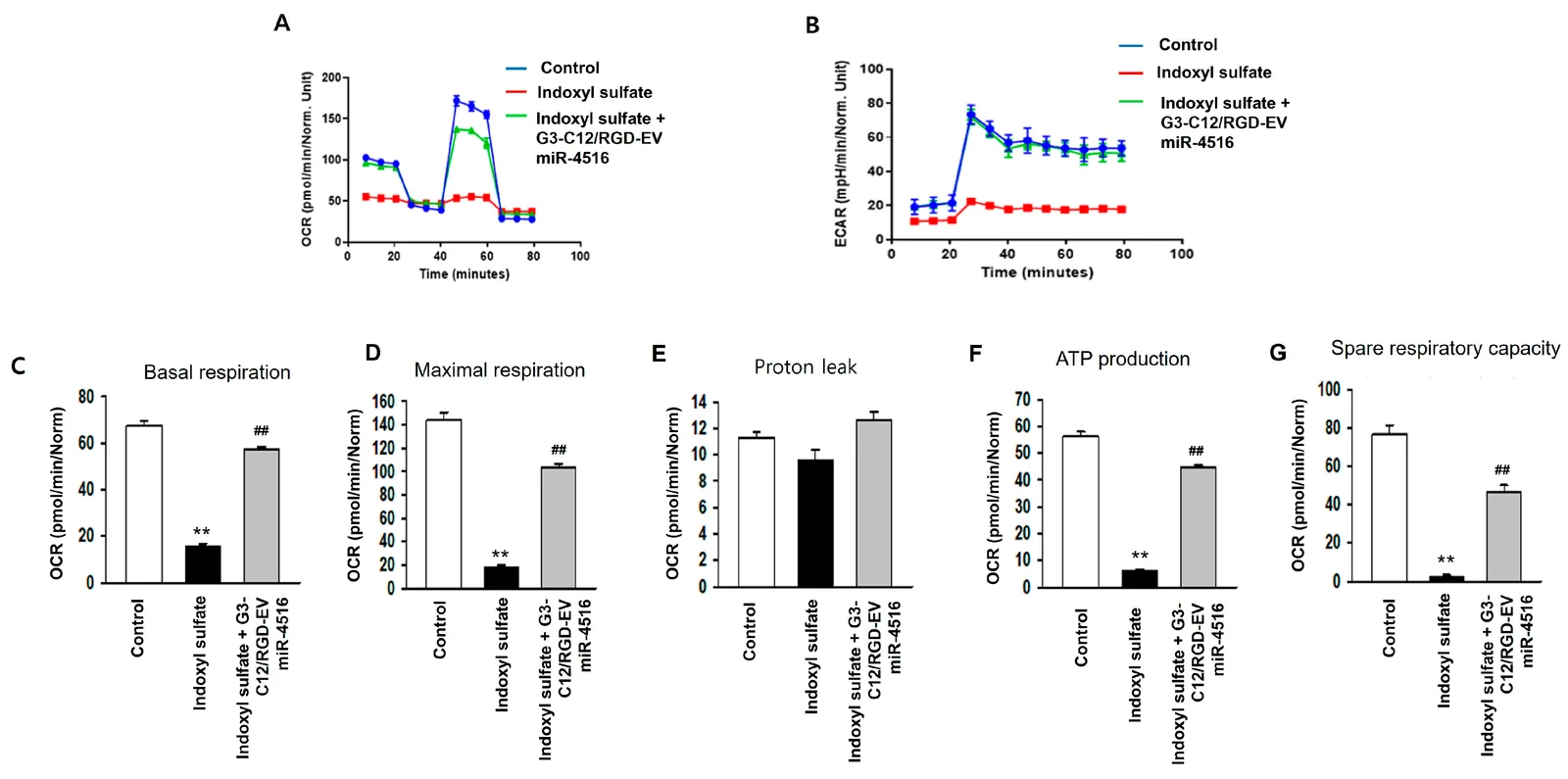
Seahorse extracellular flux analysis of OCR and ECAR in indoxyl sulfate-treated TH1 proximal tubule cells following treatment with miR-4516-loaded G3-C12/RGD-engineered EVs. TH1 proximal tubule cells were assigned to Control, Indoxyl sulfate, and Indoxyl sulfate + miR-4516-loaded G3-C12/RGD-engineered EVs (G3-C12/RGD-EV miR-4516). OCR and ECAR were measured using a Seahorse mitochondrial stress test protocol as described in Methods. (**A**) OCR trace (pmol/min/Norm) over time. (**B**) ECAR trace (mpH/min/Norm) over time. (**C**–**G**) OCR-derived parameters: (**C**) Basal respiration, (**D**) Maximal respiration, (**E**) Proton leak, (**F**) ATP production (ATP-linked respiration), and (**G**) Spare respiratory capacity. Data are presented as mean ± SEM (n = 3). Statistical analysis was performed by one-way ANOVA followed by Tukey’s post hoc test. ** *p* < 0.01 vs. Control; ## *p* < 0.01 vs. Indoxyl sulfate, as indicated in the panels.

Seahorse extracellular flux analysis was performed to assess oxygen consumption rate (OCR) and extracellular acidification rate (ECAR) in TH1 cells treated with indoxyl sulfate with or without miR-4516-loaded G3-C12/RGD-engineered EVs (Figure 4). Indoxyl sulfate markedly reduced OCR throughout the assay, whereas G3-C12/RGD-EV miR-4516 increased OCR relative to indoxyl sulfate alone (Figure 4A). Consistently, indoxyl sulfate significantly decreased basal respiration, maximal respiration, ATP production (ATP-linked respiration), and spare respiratory capacity, and these OCR-derived parameters were partially restored by G3-C12/RGD-EV miR-4516 (Figure 4C,D,F,G). In contrast, proton leak (Figure 4E) was not significantly altered among groups. ECAR showed a similar pattern, decreasing with indoxyl sulfate and increasing after G3-C12/RGD-EV miR-4516 treatment (Figure 4B).

### 2.5. miR-4516-Loaded G3-C12/RGD-Engineered EVs Reduce Indoxyl Sulfate-Induced Apoptosis in TH1 Proximal Tubule Cells

**Figure 5:**
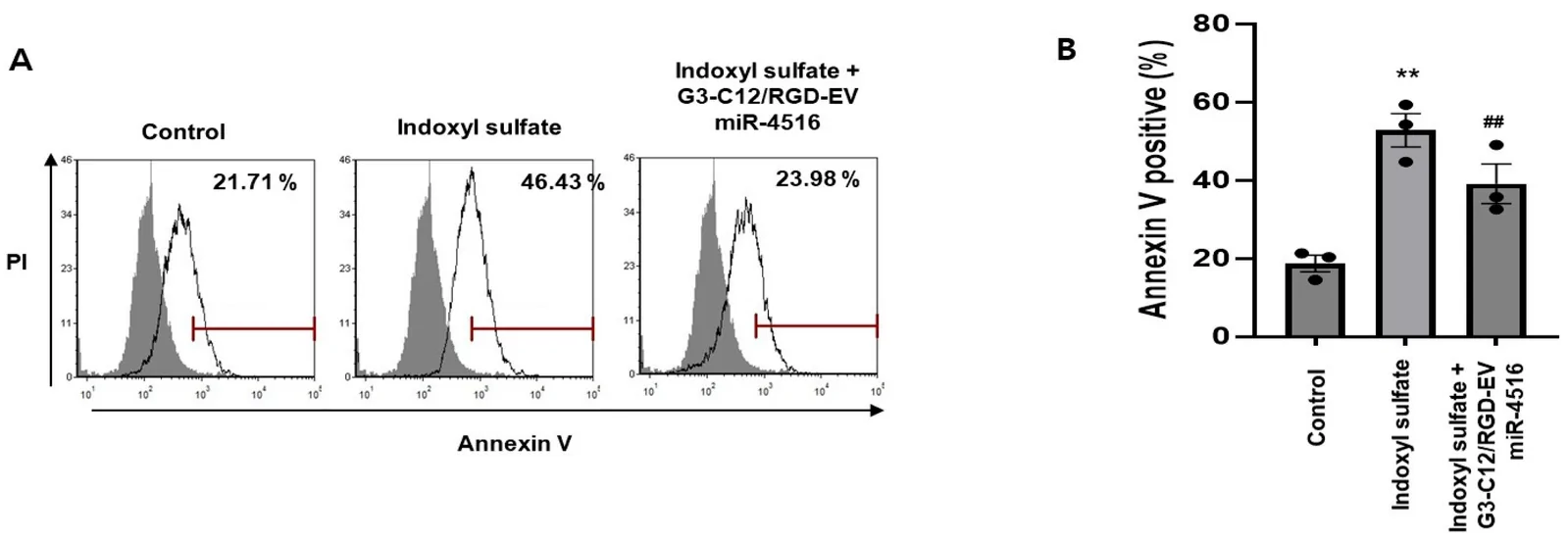
Annexin V/PI flow cytometry analysis of apoptosis in indoxyl sulfate-treated TH1 proximal tubule cells following treatment with miR-4516-loaded G3-C12/RGD-engineered EVs. TH1 proximal tubule cells were treated with Control, indoxyl sulfate, or indoxyl sulfate + miR-4516-loaded G3-C12/RGD-engineered EVs (G3-C12/RGD-EV miR-4516; preparation described in Methods) and then stained with Annexin V and propidium iodide (PI) for flow cytometry. (**A**) Representative flow cytometry plots showing the Annexin V-positive (%) population under each condition. (**B**) Quantification of Annexin V-positive cells (%). Data are presented as mean ± SEM (n = 3). Statistical analysis was performed by one-way ANOVA followed by Tukey’s post hoc test. ** *p* < 0.01 vs. Control; ## *p* < 0.01 vs. Indoxyl sulfate, as indicated in the panel.

To determine whether engineered EV treatment modulates toxin-induced cell death, Annexin V/propidium iodide (PI) staining followed by flow cytometry was performed in TH1 proximal tubule cells (Figure 5). Indoxyl sulfate increased the fraction of Annexin V-positive cells compared with the control condition, whereas treatment with miR-4516-loaded G3-C12/RGD-engineered EVs reduced Annexin V positivity relative to indoxyl sulfate alone. Representative plots showed Annexin V-positive fractions of 21.71% (Control), 46.43% (Indoxyl sulfate), and 23.98% (Indoxyl sulfate + G3-C12/RGD-EV miR-4516) (Figure 5A), consistent with the quantification in Figure 5B.

### 2.6. Intravenous Administration of miR-4516-Loaded G3-C12/RGD-Engineered EVs Improves Renal Function Indices in CKD Mice

**Figure 6:**
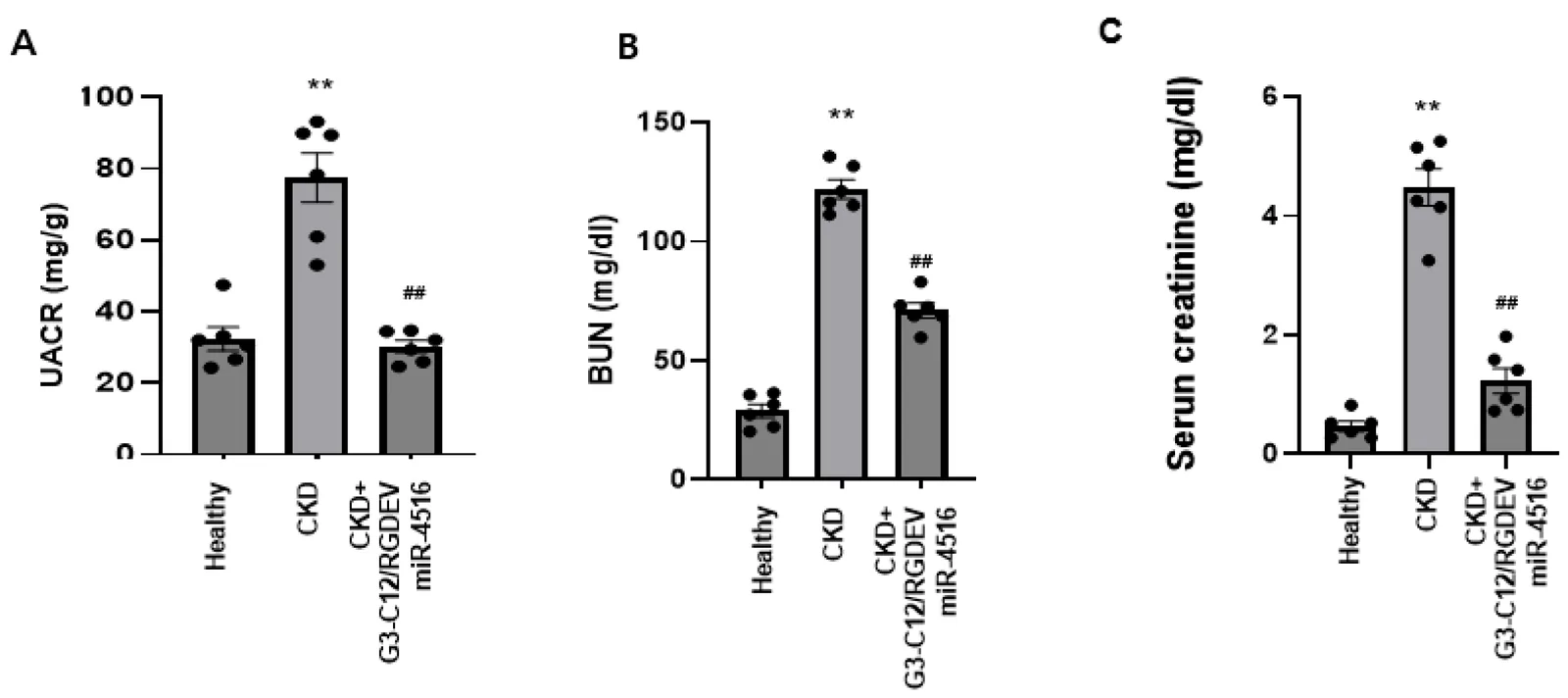
Renal function assessment in CKD mice following intravenous administration of miR-4516-loaded G3-C12/RGD-engineered EVs. Mice were grouped as Healthy, CKD, and CKD + miR-4516-loaded G3-C12/RGD-engineered EVs (G3-C12/RGD-EV miR-4516), and engineered EVs were administered by intravenous injection as described in Methods. (**A**) Urinary albumin-to-creatinine ratio (UACR), calculated as albumin (µg/mL) × 100/urine creatinine (mg/dL). (**B**) Blood urea nitrogen (BUN) (mg/dL). (**C**) Serum creatinine (mg/dL). Dots represent individual animals and bars indicate mean ± SEM. Statistical analysis was performed by one-way ANOVA followed by Tukey’s post hoc test. ** *p* < 0.01 vs. Healthy; ## *p* < 0.01 vs. CKD, as indicated in the panels.

**Figure 7:**
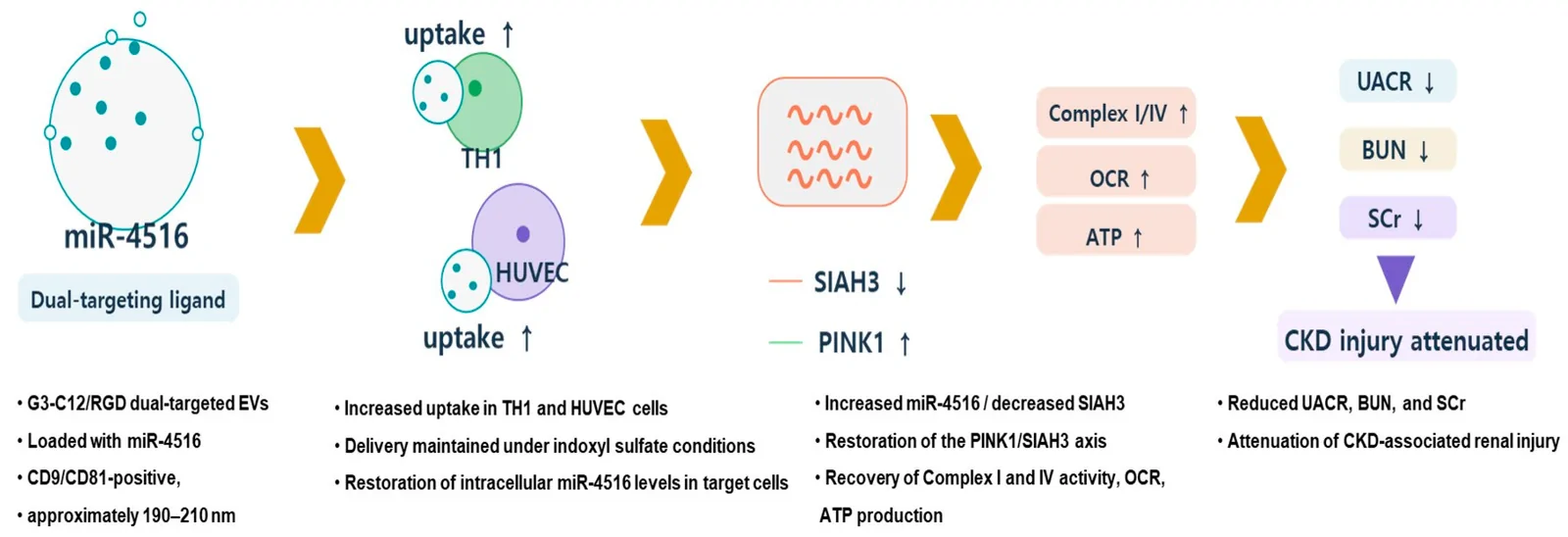
Proposed mechanism of miR-4516-loaded G3-C12/RGD-engineered extracellular vesicles in CKD. miR-4516-loaded G3-C12/RGD-engineered extracellular vesicles (EVs) showed increased cellular association/uptake in TH1 proximal tubular cells and HUVECs under indoxyl sulfate conditions. Restoration of intracellular miR-4516 was associated with decreased SIAH3 expression and increased PINK1 expression, leading to improved mitochondrial function, including increased Complex I/IV activity, oxygen consumption rate (OCR), and ATP production. In vivo, systemic administration of engineered EVs was associated with reduced urinary albumin-to-creatinine ratio (UACR), blood urea nitrogen (BUN), and serum creatinine (SCr), indicating attenuation of CKD-associated renal injury.

To evaluate whether systemic delivery of engineered EVs improves renal dysfunction in vivo, CKD mice were administered miR-4516-loaded G3-C12/RGD-engineered EVs via intravenous injection (Figure 6). CKD mice exhibited increased albuminuria compared with Healthy controls, as reflected by elevated UACR, whereas G3-C12/RGD-EV miR-4516 treatment reduced UACR relative to untreated CKD mice (Figure 6A). CKD also increased circulating markers of renal impairment, including blood urea nitrogen (BUN) and serum creatinine, and both parameters were decreased following G3-C12/RGD-EV miR-4516 administration (Figure 6B,C). These results indicate that intravenous delivery of miR-4516 via targeting-engineered EVs is associated with improvement of renal functional indices in CKD mice. Taken together, the overall findings support a model in which miR-4516-loaded G3-C12/RGD-engineered EVs enhance uptake in target cells, suppress SIAH3, restore PINK1-associated mitochondrial function, and thereby attenuate CKD-associated renal injury (Figure 7).

## 3. Discussion

Chronic kidney disease (CKD) progresses through persistent tubular and interstitial injury accompanied by maladaptive repair responses that shift the renal cortex toward fibrotic remodeling and functional decline. In parallel with fibrosis, microvascular rarefaction and endothelial dysfunction aggravate tissue hypoxia and oxidative stress, reinforcing tubulointerstitial damage and limiting recovery. This pathophysiologic coupling supports therapeutic concepts that consider both tubular and vascular compartments rather than focusing on a single cell population [16,17].

MicroRNAs (miRNAs) regulate gene expression through sequence-specific interactions with target mRNAs and have been implicated in pathways relevant to CKD progression, including fibrogenic signaling and cellular stress responses [18]. In prior studies, miR-4516 was reduced in CKD-related settings, and melatonin-associated restoration of miR-4516 coincided with attenuation of renal cortical fibrosis and improvement of mitochondrial homeostasis, supporting miR-4516 as a regulator linked to renal injury phenotypes [10,19]. Building on this background, the present study evaluated a direct miRNA replacement strategy using engineered extracellular vesicles (EVs) to deliver miR-4516 to proximal tubular epithelial cells and vascular endothelial cells. In this study, EV cellular association/uptake was quantified as the fraction of CMO-positive cells and was interpreted as reflecting cellular association/uptake under the experimental conditions. Functional delivery of the RNA cargo was evaluated in parallel by cellular restoration of miR-4516 and by changes in mitochondrial bioenergetics and apoptosis readouts under uremic toxin stress.

To implement miR-4516 replacement, we selected milk-derived EVs as the carrier, given prior evidence supporting their use as biocompatible vesicular platforms for nucleic acid transport [20,21,22]. In addition, our previous work established an isolation and engineering workflow for bovine milk EVs, including density-gradient purification, physicochemical characterization, and chemical surface conjugation for functionalization, supporting feasibility for reproducible production and downstream engineering steps [23]. Together, these features support consideration of milk EVs as a supply source in preclinical development, where batch scale and handling stability can be limiting factors [20,21,22].

At the same time, the present study does not support definitive subtype classification of these vesicles as exosomes. Accordingly, we use the broader term extracellular vesicles (EVs) throughout the manuscript. In addition, although the preparations were characterized by density-gradient purification, nanoparticle tracking analysis, and immunoblotting for CD9 and CD81, broader purity assessment, including protein-to-particle ratio, additional positive markers such as TSG101 and ALIX, negative or depleted markers, and evaluation of possible co-isolated milk contaminants such as casein or lipoproteins, was not comprehensively performed in the present study. These points should be addressed in future studies to strengthen vesicle identity and purity assessment.

To increase cellular association/uptake in tubular and endothelial compartments, EV surfaces were functionalized with an RGD-containing targeting moiety prior to miR-4516 loading. EV surface engineering has been used to augment tissue or cell-type association by leveraging ligand–receptor interactions, including integrin-mediated binding of RGD motifs [11,24,25,26]. The increased cellular association/uptake observed in both proximal tubular cells and endothelial cells is consistent with the premise that integrin-related interactions can increase effective cellular association and, in turn, improve intracellular exposure to RNA cargo. Given the broad distribution of RGD-binding integrins, quantitative biodistribution and target-engagement analyses will be required to define tissue- and cell-level delivery under systemic dosing [11,25,26].

The functional relevance of engineered EV delivery was evaluated under indoxyl sulfate exposure. Indoxyl sulfate is a clinically relevant, protein-bound uremic toxin that accumulates in CKD and contributes to tubular injury and broader organ dysfunction through oxidative stress and profibrotic signaling [27]. Prior studies have linked protein-bound uremic toxins, including indoxyl sulfate, to mitochondrial injury, altered mitochondrial dynamics, and impaired cellular homeostasis in renal contexts [28,29]. In the present study, indoxyl sulfate reduced indices of mitochondrial respiratory chain activity and depressed mitochondrial respiration, whereas engineered EV-mediated miR-4516 delivery partially restored respiratory function and improved respiration-related flux parameters. These findings extend miR-4516-associated observations to a direct RNA delivery setting and are consistent with the interpretation that miR-4516 restoration can mitigate uremic toxin-associated mitochondrial impairment [18,29].

In parallel, miR-4516 delivery reduced toxin-induced apoptosis, as indicated by decreased Annexin V positivity in indoxyl sulfate-treated cells. This pattern aligns with the established coupling between mitochondrial energetic failure and intrinsic apoptotic signaling during cellular stress. While the present results indicate phenotypic recovery at the level of mitochondrial respiration and apoptosis, additional mechanistic mapping will be needed to connect miR-4516 replacement to defined quality-control pathways that govern mitochondrial integrity in tubular injury. In this regard, prior work implicating the miR-4516/SIAH3/PINK1 axis and mitophagy-related signaling provides a framework for follow-up studies in the context of EV-mediated replacement [19].

Consistent with the in vitro findings, systemic administration of miR-4516-loaded engineered EVs improved renal functional indices in CKD mice, including reduced albuminuria and improved circulating markers of renal impairment. Concordant directionality across functional measures supports the biological relevance of the approach; however, functional improvement alone does not define the dominant anatomic site or pathway responsible for benefit. Future studies integrating tissue-level miR-4516 enrichment, cell-type-resolved delivery readouts, and pathway profiling would strengthen interpretation of kidney/endothelium-directed effects under systemic dosing [11,25,26]. In addition, transcript- and protein-level profiling will be important to confirm on-pathway engagement while surveying potential off-pathway effects, given the multi-target nature of miRNAs [18]. In addition, the translational applicability of milk-derived EVs depends not only on biological efficacy but also on practical manufacturing considerations. Although milk is an attractive and accessible source for EV isolation, batch-to-batch variability, the effects of upstream processing such as pasteurization, storage stability, and the feasibility of large-scale production may influence EV yield, composition, and functional consistency. These variables may limit reproducibility and translational scalability unless source material, purification workflow, storage conditions, and quality-control parameters are further standardized. Therefore, further standardization of source material, purification workflow, storage conditions, and quality-control parameters will be necessary for future preclinical and translational development. In this context, the present study should be regarded as a proof-of-concept evaluation of therapeutic potential rather than a fully optimized manufacturing platform. Recent studies have further highlighted the therapeutic and translational potential of milk-derived EVs as bioactive carriers and drug delivery platforms [30,31]. At the same time, accumulating evidence indicates that broader preclinical and translational application will require more careful consideration of isolation strategy, vesicle heterogeneity, storage stability, and scalable production [31,32,33]. These issues should be addressed in future studies to support the development of engineered milk-derived EVs as a more robust and clinically relevant delivery platform [31,32,33].

Finally, dose–response relationships, durability of effect, and repeat-dose safety, particularly in the setting of surface modification, remain key translational considerations for milk-derived EV-based miRNA replacement [11,20,25]. In addition, the present study has several limitations. Histopathological evaluation of major organs was not available. Therefore, although the current findings support the therapeutic potential of miR-4516-loaded G3-C12/RGD-engineered EVs, more comprehensive safety assessment, including major-organ histopathology and systemic toxicity studies, will be required in future preclinical investigations. In addition, the localization of loaded miR-4516 within EV preparations was not directly validated by RNase protection assays performed in the presence or absence of detergent. Although RT–qPCR analysis demonstrated significant enrichment of miR-4516 in re-purified EV preparations after loading, the present data do not definitively distinguish intravesicular encapsulation from externally associated miRNA. Therefore, the current findings support EV-associated enrichment of miR-4516 after loading, but do not by themselves establish intravesicular localization or fully define the EVs as delivery vehicles in a strict mechanistic sense. Additional studies will therefore be required to confirm the localization and protection status of the loaded miRNA.

In summary, the present study supports an engineered EV approach to deliver miR-4516 to tubular epithelial and endothelial compartments, thereby attenuating uremic toxin-associated mitochondrial dysfunction and apoptosis in vitro and improving renal functional indices in a CKD mouse model. These findings indicate that miR-4516 replacement delivered by engineered milk-derived EVs warrants further evaluation with aligned biodistribution, target engagement, and safety endpoints.

## 4. Materials and Methods

### 4.1. Cell Culture

Human renal proximal tubule epithelial TH1 cells were purchased from Kerafast (Boston, MA, USA; Cat. No. ECH001; Cellosaurus accession no. CVCL_5J51; RRID: CVCL_5J51). TH1 cells were cultured in minimum essential medium (MEM; Gibco BRL, Gaithersburg, MD, USA) supplemented with 10% (v/v) fetal bovine serum (FBS; Gibco BRL, Grand Island, NY, USA) and 100 U/mL penicillin/streptomycin (Gibco BRL, Grand Island, NY, USA). Human umbilical vein endothelial cells (HUVECs) were obtained from the American Type Culture Collection (ATCC, Manassas, VA, USA) and maintained in endothelial cell growth medium (EGM-2; Lonza, Walkersville, MD, USA) supplemented with the EGM-2 BulletKit components and 100 U/mL penicillin/streptomycin. Culture vessels for HUVECs were pre-coated with 0.1% gelatin (*w*/*v*) at 37 °C for 30 min or fibronectin (10 μg/mL; Sigma-Aldrich, St. Louis, MO, USA) at 37 °C for 1 h before cell seeding. All cells were grown at 37 °C in a humidified incubator with 5% CO2, and the medium was replaced every 2 days. Cells were passaged at 70–80% confluence using 0.05% trypsin–EDTA (Gibco BRL, Grand Island, NY, USA) and used for experiments at passages 3–7.

### 4.2. Mitochondrial Fraction

Preparation: To evaluate mitochondrial functions, we harvested TH1 cells and isolated their mitochondria using a mitochondria fraction kit (Thermo Fisher Scientific, Waltham, MA, USA). Isolated mitochondria were subsequently incubated for 10 min on ice. The cell lysates with isolation buffer were centrifuged at 700× *g* for 10 min at 4 °C. Supernatant was collected and centrifuged again at 12,000× *g* for 15 min at 4 °C. After washing with fraction buffer, the residual pellet was lysed with RIPA lysis buffer and then centrifuged at 12,000× *g* for 30 min at 4 °C.

### 4.3. Isolation and Purification of Milk-Derived Extracellular Vesicles (EVs)

Milk-derived extracellular vesicles (EVs) were isolated from commercially available pasteurized bovine milk by sequential differential centrifugation followed by iodixanol density-gradient ultracentrifugation, as previously described with minor modifications [12]. Briefly, milk was centrifuged at 2000× *g* for 10 min and 10,000× *g* for 40 min to remove fat and debris. The resulting supernatant was further centrifuged at 35,000× *g* for 1 h. EVs were then pelleted by ultracentrifugation at 100,000× *g* for 1 h, resuspended in phosphate-buffered saline (PBS), and subjected to density-gradient purification. A discontinuous iodixanol step gradient was prepared using 50% (*w*/*v*) and 10% (*w*/*v*) iodixanol (Sigma-Aldrich, St. Louis, MO, USA). The 50% iodixanol solution was layered at the bottom of the ultracentrifuge tube, followed by the 10% iodixanol solution and the PBS-resuspended EV pellet. After ultracentrifugation at 100,000× *g* for 1 h, the EV-enriched fraction at the interface between 10% and 50% iodixanol was collected and used for subsequent experiments. All centrifugation steps were performed at 4 °C. The isolated EV preparations were characterized by nanoparticle tracking analysis (NTA) and immunoblotting for the EV markers CD9 and CD81, as described below. The experiments described in the present study were performed using independent EV preparations generated by this workflow. For miRNA loading and quantification experiments, EV input was normalized by particle number determined by NTA prior to downstream analyses.

### 4.4. Display of G3-12 and RGD Peptide on the Surface of Milk-Derived Extracellular Vesicles (EVs)

A total of 900 μg of milk EVs was mixed with 300 μg cholesterol-PEG-DBCO and incubated for 1 h at room temperature. DBCO-incorporated milk EVs were isolated by iodixanol density-gradient ultracentrifugation using a 10–50% step gradient. The DBCO-incorporated milk EVs were then mixed with a total of 200 μg of azide-modified peptides (G3-C12 and RGD) and incubated for 1 h at room temperature. G3-C12/RGD-displayed milk EVs were isolated again by iodixanol density-gradient ultracentrifugation using a 10–50% step gradient. Control milk EVs were processed in parallel under the same conditions without cholesterol-PEG-DBCO and peptides. Cholesterol-PEG-DBCO was purchased from Nanocs (New York, NY, USA). G3-C12 and RGD peptides were synthesized by Peptron (Daejeon, Republic of Korea) (G3-C12 peptide sequence: Azidopentanoyl-GGGSANTPCGPYTHDCPVKR; RGD peptide sequence: Cyclo[RGDyK(Azidopentanoyl)]).

### 4.5. Milk-Derived Extracellular Vesicles (EVs) Labeling and Uptake Analysis

Extracellular vesicles (EVs) were fluorescently labeled by incubation with CellMask™ Orange (CMO) plasma membrane stain (Sigma-Aldrich) at 5 μg/mL for 1 h at room temperature. Unbound dye was removed, and CMO-labeled EVs were recovered by density-gradient ultracentrifugation. TH1 cells and HUVECs were incubated with CMO-labeled control EVs or CMO-labeled G3-C12/RGD-EVs loaded with miR-4516 for 2 h at 37 °C. After incubation, cells were washed with PBS. For confocal microscopy, cells were stained with CFDA (green) and counterstained with DAPI (blue), and images were acquired using a confocal laser scanning microscope (Leica, Wetzlar, Germany). For flow cytometric quantification, cells were prepared as single-cell suspensions and analyzed using a CyFlow^®^ Cube 8 flow cytometer (Sysmex Partec, Görlitz, Germany). Gates were set based on non-treated cells as the negative control, and EV cellular association/uptake was expressed as the percentage of CMO-positive cells.

### 4.6. miR-4516 Loading into Engineered Milk-Derived Extracellular Vesicles (EVs)

Purified milk-derived EVs were first surface-engineered with G3-C12 and RGD to generate G3-C12/RGD-EVs, and the engineered EVs were subsequently loaded with synthetic miR-4516 using the Exo-Fect™ siRNA/miRNA Transfection Kit (System Biosciences, Palo Alto, CA, USA) according to the manufacturer’s instructions. Synthetic miR-4516 was added at the indicated input amounts (150 or 300 μg) during the loading reaction. After loading, EVs were re-isolated to remove unincorporated miRNA and residual reagents, and the resulting preparations were used as miR-4516-loaded G3-C12/RGD-EVs for subsequent analyses.

### 4.7. Quantification of Milk-Derived Extracellular Vesicles (EVs)-Loaded miRNA-4516

Total RNA was isolated from engineered EV preparations (Control EVs and miR-4516-loaded G3-C12/RGD-EVs) using the mirVana™ miRNA Isolation Kit (Thermo Fisher Scientific, Waltham, MA, USA) according to the manufacturer’s instructions. EV input was normalized by particle number determined by nanoparticle tracking analysis (NTA) prior to RNA extraction. An exogenous spike-in miRNA (cel-miR-39) was added to each sample before RNA isolation to control for extraction and reverse transcription efficiency. cDNA was synthesized using a poly(A) tailing method (Applied Biological Materials, Richmond, BC, Canada), and qRT-PCR was performed using the TaqMan™ Small RNA Assay (Thermo Fisher Scientific). miR-4516 levels were normalized to the spike-in control and are presented as fold change relative to Control EVs.

### 4.8. Western Blot Analysis

Protein extracts of milk-derived extracellular vesicles (EVs) were separated by 10–15% sodium dodecyl sulfate–polyacrylamide gel electrophoresis (SDS-PAGE) and then transferred to a 0.2 µm PVDF membrane. Blocking was performed with 3% skim milk or a protein-free blocking buffer (Thermo Fisher Scientific, Waltham, MA, USA) for 1 h. The membrane was incubated with primary antibodies against CD81 (1:1000; SC-166029, Santa Cruz Biotechnology, Dallas, TX, USA) and CD9 (1:1000; NB500-494, Novus Biologicals, Littleton, CO, USA) PINK1 (1:1000; Santa Cruz Biotechnology, Dallas, TX, USA, sc-517353), SIAH3 (1:1000; Novus, Littleton, CT, USA, NBP2-83524), VDAC1 (1:1000; Novus, NB100-695) for 2 h at room temperature (RT). After washing with Tris-buffered saline containing 0.05% Tween-20 (TBS-T), the membrane was incubated with a horseradish peroxidase (HRP)-conjugated goat anti-mouse IgG secondary antibody (Santa Cruz Biotechnology). Bands were detected using enhanced chemiluminescence (ECL; Amersham Biosciences, Uppsala, Sweden).

### 4.9. Nanoparticle Tracking Analysis of Milk-Derived Extracellular Vesicles (EVs)

The particle concentration and size distribution of milk EVs were determined by nanoparticle tracking analysis (NTA) using a NanoSight LM10-HS system (Malvern Instruments Ltd., Malvern, UK) equipped with a 688 nm laser. Data were analyzed using NTA software (version 2.3; Malvern Instruments Ltd.).

### 4.10. Mitochondrial Complex I and IV Activity

Mitochondrial fractions were isolated from TH1 cells and used for complex respiratory activity assays. Complex I activity was measured using the Complex I Enzyme Activity Microplate Assay Kit (ab109721; Abcam, Cambridge, UK) according to the manufacturer’s instructions. Complex IV activity was measured using the Complex IV Activity Assay Kit (Rodent) (ab109911; Abcam, Cambridge, UK) according to the manufacturer’s instructions. Activities were normalized to mitochondrial protein content and are presented as ΔOD/min/mg.

### 4.11. OCR Measurements and Calculation of Bioenergetic Parameters

Mitochondrial respiration and glycolytic activity were assessed using an XF96 Extracellular Flux Analyzer (Agilent Technologies, Santa Clara, CA, USA). TH1 cells were seeded in XF96 cell culture microplates at 45,000 cells/well in growth medium and incubated for 24 h at 37 °C in a humidified atmosphere containing 5% CO_2_. Cells were treated with vehicle (control) or indoxyl sulfate and, where indicated, additionally treated with miR-4516-loaded G3-C12/RGD-engineered EVs prior to Seahorse analysis (Figure 4A–G). After the indicated treatments, cells were washed and equilibrated in XF assay medium (Agilent Technologies) supplemented with 25 mM glucose and incubated for 30 min at 37 °C in a non-CO_2_ incubator. The oxygen consumption rate (OCR) and extracellular acidification rate (ECAR) were recorded in cycles consisting of 3 min mixing, 2 min waiting, and 2 min measurement, and values were collected at 7 min intervals using Seahorse Wave Desktop Software version 2.6 (Agilent Technologies, Santa Clara, CA, USA). 

For the Mito Stress Test, OCR was measured under basal conditions and after sequential injections of oligomycin (1.5 μM), FCCP (1.0 μM), and rotenone/antimycin A (0.5 μM), with three measurement cycles performed after each injection. Bioenergetic parameters were calculated from OCR traces as follows: basal respiration was obtained by subtracting non-mitochondrial respiration (OCR remaining after rotenone/antimycin A) from baseline OCR prior to oligomycin; maximal respiration was defined as OCR after FCCP injection minus non-mitochondrial respiration; ATP-linked respiration (ATP production) was calculated as the difference between baseline OCR and OCR after oligomycin; and spare respiratory capacity was calculated as maximal respiration minus basal respiration. ECAR was monitored in parallel and presented as an index of glycolytic activity. After Seahorse measurements, cells were lysed and total protein content per well was determined using a BCA assay. OCR and ECAR values were normalized to total protein content per well and are presented as pmol/min/Norm and mpH/min/Norm.

### 4.12. Apoptosis Analysis by Flow Cytometry

Apoptosis was assessed by Annexin V–FITC/propidium iodide (PI) staining followed by flow cytometry. Cells were treated with PBS (vehicle control), indoxyl sulfate, or indoxyl sulfate plus miR-4516-loaded G3-C12/RGD-EVs, as indicated. After treatment, cells were collected, washed with PBS, and stained with Annexin V–FITC and PI (Sigma-Aldrich) according to the manufacturer’s instructions. Fluorescence signals were acquired using a CyFlow Cube 8 flow cytometer (Sysmex Partec, Münster, Germany), and data were analyzed using FCS Express software (De Novo Software, Pasadena, CA, USA). Apoptosis was quantified as the percentage of Annexin V-positive cells.

### 4.13. In Vivo CKD Mouse Model and Renal Function Assessment

Eight-week-old male BALB/c mice were fed an adenine-containing diet (0.25% adenine) for 3 weeks, and body weights were monitored weekly. Mice were randomly assigned to three groups (n = 6 per group): Healthy, CKD + PBS, and CKD + miR-4516-loaded G3-C12/RGD-EVs. CKD was induced by adenine feeding, whereas the Healthy group received a standard diet. To evaluate the therapeutic effect of engineered EVs, mice in the CKD groups received PBS or miR-4516-loaded G3-C12/RGD-EVs via tail-vein injection beginning after 7 days of adenine feeding. EVs were administered at a dose of 1 × 10^10^ particles per mouse per injection. Injections were given twice per week (every 3–4 days) for 2 weeks (a total of 4 injections). Particle numbers were determined by nanoparticle tracking analysis (NTA). For urinary biomarker assessment, mice were housed in 24 h metabolic cages to collect urine. Urinary albumin and creatinine were measured, and the urinary albumin-to-creatinine ratio (UACR) was calculated as albumin (µg/mL) × 100/urine creatinine (mg/dL). At the endpoint, blood samples were collected and stored at −80 °C until analysis. Serum BUN and creatinine were measured using commercial kits (BUN: MYBioSource (San Diego, CA, USA); creatinine: Crystal Chem (Elk Grove Village, IL, USA)).

## 5. Conclusions

This study establishes an engineered Milk-derived extracellular vesicle (EV)-based miR-4516 supplementation approach using milk-derived extracellular vesicles functionalized with G3-C12/RGD and loaded with miR-4516. In indoxyl sulfate-treated proximal tubule cells, EV-mediated miR-4516 delivery increased intracellular miR-4516 levels and was associated with improved mitochondrial function, as indicated by increased respiratory chain Complex I and Complex IV activities and improved Seahorse bioenergetic parameters. In an adenine diet-induced CKD mouse model, intravenous administration of miR-4516-loaded engineered EVs was associated with improved renal functional indices, including UACR, BUN, and serum creatinine. Collectively, these findings suggest that engineered EV-mediated miR-4516 delivery may mitigate uremic toxin-associated mitochondrial dysfunction and renal impairment in CKD.

## Data Availability

The data presented in this study are available from the corresponding author, S.H.L., upon reasonable request.
